# Signal Quality Assessment and Reconstruction of PPG-Derived Signals for Heart Rate and Variability Estimation in In-Vehicle Applications: A Comparative Review and Empirical Validation

**DOI:** 10.3390/s25247556

**Published:** 2025-12-12

**Authors:** Ruimin Gao, Carl S. Miller, Brian T. W. Lin, Chris W. Schwarz, Monica L. H. Jones

**Affiliations:** 1 School of Psychology, Georgia Institute of Technology, Atlanta, GA 30332, USA; 2 Transportation Research Institute, College of Engineering, University of Michigan, Ann Arbor, MI 48109, USA; 3 Driving Safety Research Institute, College of Engineering, University of Iowa, Iowa City, IA 52242, USA

**Keywords:** photoplethysmography (PPG), electrocardiography (ECG), heart rate (HR), heart rate variability (HRV), signal quality assessment, wearable sensors, in-vehicle monitoring, motion artifacts, signal reconstruction

## Abstract

Electrocardiography (ECG) is widely recognized as the gold standard for measuring heart rate (HR) and heart rate variability (HRV). However, photoplethysmography (PPG) presents notable advantages in terms of wearability, affordability, and ease of integration into consumer devices, despite its susceptibility to motion artifacts and the absence of standardized processing protocols. In this study, we review current ECG and PPG signal processing methods and propose a signal quality assessment and reconstruction pipeline tailored for dynamic, in-vehicle environments. This pipeline was evaluated using data gathered from participants riding in an automated vehicle. Our findings demonstrate that while blood volume pulse (BVP) derived from PPG can provide reliable heart rate estimates and support extraction of certain HRV features, its utility in accurately capturing high-frequency HRV components remains constrained due to motion-induced noise and signal distortion. These results underscore the need for caution in interpreting PPG-derived HRV, particularly in mobile or ecologically valid contexts, and highlight the importance of establishing best practices and robust preprocessing methods to enhance the reliability of PPG sensing for field-based physiological monitoring.

## 1. Introduction

Cardiovascular health is a vital indicator of human well-being, with heart rate and its variability widely used to assess autonomic nervous system function. In clinical contexts, electrocardiogram (ECG or EKG) monitoring enables the detection of arrhythmias, myocardial infarctions, and other cardiac abnormalities [1]. Beyond clinical settings, heart rate (HR) and heart rate variability (HRV) have been used to assess stress, fatigue, and cognitive workload in in-field applications, particularly in driving, human factors, and mobile work environments [2].

While ECG remains the gold standard for cardiac monitoring, the growing availability of light-based photoplethysmography (PPG) technology has enabled wearable solutions, such as wristbands and finger-clip sensors, that estimate cardiovascular metrics by detecting blood volume pulse (BVP) changes in the skin. These PPG systems offer practical advantages including comfort, affordability, and ease of use, and have been increasingly adopted in applied research contexts, such as detection of driver drowsiness [3], driver mental fatigue [4], and passenger ride comfort [5]. Despite these benefits, PPG presents several limitations. Compared to ECG, it is more susceptible to motion artifacts [6], skin tone variability [7], and inconsistent sensor contact pressure [6,8]. In addition, the lack of standardized hardware configurations, acquisition protocols, and signal processing pipelines introduces variability that undermines reproducibility and generalizability [9]. Although pulse rate estimates derived from PPG are generally accurate in healthy individuals at rest, questions remain regarding their validity as a surrogate for ECG-derived HRV, especially in dynamic or ecologically relevant conditions typical of in-field applications. Recent advances in machine learning-assisted point-of-care diagnostics further illustrate the movement toward real-time, automated cardiovascular monitoring [10].

To address this gap, the present study pursues three primary objectives. First, we review the existing literature on ECG and PPG signal characteristics and outline emerging trends in signal processing methods relevant to wearable and mobile sensing. Second, we evaluate the effectiveness of current BVP processing techniques for cardiovascular tracking using a dataset gathered from participants during an in-vehicle field study. Finally, we provide practical recommendations and highlight key considerations for implementing and refining PPG-based monitoring pipelines for in-vehicle and other dynamic applications.

### 1.1. ECG and PPG Processing: Previous Research

#### 1.1.1. ECG

ECG measures the electrical activity generated by the depolarization and repolarization of the heart muscle during each cardiac cycle [11]. In clinical and laboratory settings, ECG signals are typically acquired using electrodes placed on the chest and limbs to ensure high-fidelity recordings [12]. For ambulatory and activities-of-daily-living monitoring, portable ECG devices such as Holter monitors allow continuous tracking of cardiac rhythms, particularly for the detection of arrhythmias. These devices are commonly worn with chest straps; however, their bulk and prolonged skin contact can lead to discomfort or irritation [13].

**Figure 1:**
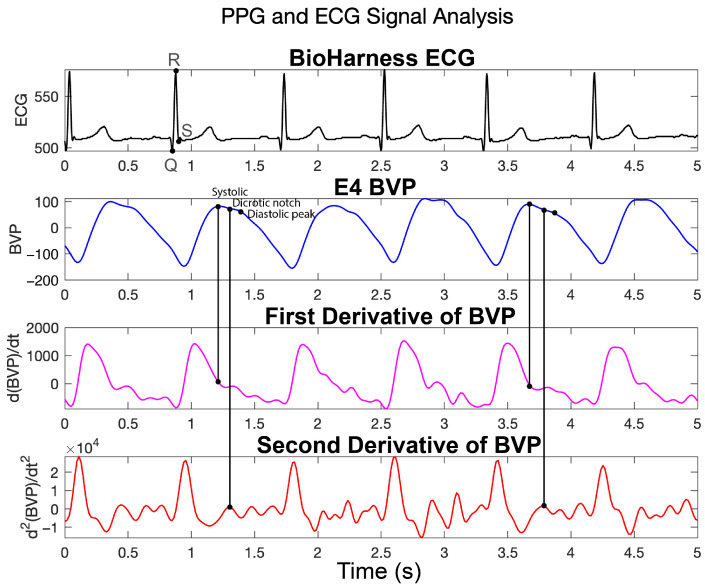
Representative ECG and PPG-derived signals from a single participant over a 5-s window. Panels show (top to bottom): BioHarness ECG waveform with annotated fiducial points, E4 BVP signal with systolic and diastolic peaks, the first derivative of the BVP (dBVP/dt), and the second derivative of the BVP (d^2^BVP/dt^2^).

Each heartbeat in the ECG waveform is characterized by a sequence of electrical events: the P-wave (atrial depolarization), QRS complex (ventricular depolarization), and T-wave (ventricular repolarization) (see Figure 1) [1]. Processing of ECG signals generally involves high-pass and low-pass filtering, fiducial point detection using established algorithms, and extraction of features for subsequent analysis [1,14,15,16,17,18,19,20].

Key ECG-derived parameters include HR and HRV. HRV provides insight into autonomic nervous system function and is typically quantified through time-domain, frequency-domain, and nonlinear (geometric and entropy-based) measures [20,21]. Time-domain metrics such as SDNN (standard deviation of all NN intervals), RMSSD (root mean square of successive differences), and pNN50 (the percentage of NN interval differences greater than 50 msec) quantify beat-to-beat interval variability. These metrics focus specifically on normal-to-normal (NN) intervals and are primarily indicative of parasympathetic modulation. Frequency-domain metrics decompose heart rate variability into spectral components: ultra-low frequency (ULF) and very-low frequency (VLF) components reflect long-term regulatory mechanisms; low-frequency (LF) power captures mixed sympathetic-parasympathetic modulation; high-frequency (HF) power is associated predominantly with parasympathetic (vagal) activity; and the LF/HF ratio is commonly interpreted as an index of sympathovagal balance, although this interpretation remains debated in the literature. Nonlinear metrics include Poincaré plot-based SD1/SD2 ratio, approximate entropy, sample entropy, multiscale entropy (MSE), and detrended fluctuation analysis (DFA) of heartbeat time series. These measures provide insights beyond linear analyses, particularly in understanding autonomic regulation under stress, fatigue, or cognitive load.

Although 5 min ECG recordings are typically recommended for robust HRV estimation, particularly for frequency-domain features like LF and HF, shorter segments may yield valid estimates under controlled conditions [21,22]. ECG signal processing techniques are well-standardized and governed by clinical guidelines issued by the American Heart Association (AHA) and the American College of Cardiology (ACC) [15]. Table 1 summarizes key ECG processing procedures and corresponding standards.

#### 1.1.2. BVP

PPG is a light-based, noninvasive technology used to measure blood volume changes in the microvascular bed of the skin. A typical PPG system includes light-emitting diodes (LEDs) and a photodetector that captures either the absorbed or reflected light, depending on tissue perfusion. PPG sensors are commonly configured in one of two ways: Transmissive PPG allows light to pass through the tissue (e.g., fingertip or earlobe) where the photodetector is positioned opposite the emitter. This configuration generally offers higher signal clarity due to its well-defined optical path. Reflective PPG, by contrast, places the emitter and detector on the same side of the tissue. Light scattered back from the underlying vasculature is captured, following a nonlinear path. While this setup is more prone to noise and motion artifacts, it is more versatile and can be applied to body sites like the wrist, while transmissive sensing is impractical [22,23]. PPG sensors are most commonly integrated into smartwatches due to their convenience and wearability [13]. However, BVP waveform quality varies with the sensor’s anatomical location. Finger-based measurements consistently yield the highest signal fidelity, outperforming those from the wrist, arm, earlobe, and forehead in terms of analyzable waveforms [24].

Unlike ECG, which features well-defined QRS complexes, BVP waveforms primarily comprise systolic and diastolic phases (see Figure 1). These are highly sensitive to external influences, such as motion, ambient light, and sensor pressure, factors that distort fiducial points (e.g., pulse onset and peak), complicating feature extraction. Fine et al. [25] reviewed sources of inaccuracy in PPG, attributing them to physiological variability, vascular characteristics, and external technical conditions. While some sources of artifact, such as ambient light and pressure inconsistencies, can be mitigated through hardware design, motion artifacts remain particularly problematic in mobile or in-vehicle contexts. Their frequency overlaps with physiological pulse rates, making them difficult to filter cleanly using traditional methods.

To date, no universal gold standard exists for BVP signal processing [9,22]. Table 2 summarizes commonly employed processing options. Preprocessing often begins with bandpass filtering and pulse detection algorithms [22]. Artifact removal methods include adaptive filters [26,27,28], signal decomposition techniques (e.g., wavelets and empirical mode decomposition) [28,29,30], and hybrid approaches [31,32,33]. More recently, deep learning models, such as CycleGANs [34] and wavelet-based neural networks [35], have been explored to reconstruct clean signals from noisy inputs.

Due to the persistent challenges in isolating motion artifacts, signal quality assessment (SQA) has emerged as a critical preprocessing step. These methods aim to distinguish clean, analyzable PPG signals from corrupted ones. Orphanidou [36] offered an early review of automated filtering and machine learning-based classification techniques. More recent studies have achieved high classification performance: Shin [35] trained a deep convolutional neural network (CNN) model that achieved an area under the curve (AUC) of 0.98 using Bayesian optimization. Mohagheghian et al. [37] proposed an ensemble-based feature selection method, yielding over 93% classification across diverse signal types. Moscato et al. [38] introduced a dual-support vector machines (SVM)-based model, with one machine optimized for heart rate estimation and the other for waveform morphology, achieving classification accuracies of 0.96 and 0.97. Despite these advancements, most models rely on expert-annotated labels, introducing subjectivity, and require large and well-curated datasets for effective training, which remain scarce in real-world in-vehicle contexts.

Once cleaned, BVP signals are often processed using techniques adapted from ECG-based HRV analysis. Accurate identification of fiducial points, including the pulse onset, systolic peak, dicrotic notch, and diastolic peak, is essential and can be achieved using methods based on derivatives, slope thresholds, wavelets, or other mathematical operators [39]. In a benchmark study, Charlton et al. [40] evaluated 15 open-source algorithms. Among them, Multi-Scale Peak and Trough Detection (MSPTD) and qppg demonstrated superior performance across diverse scenarios, including exercise, neonatal recordings, and atrial fibrillation. When time-domain fiducials are unreliable due to noise, frequency-domain alternatives (e.g., spectral peak tracking) may be used to estimate pulse rate, as showcased in the IEEE Signal Processing Cup (SPC) 2015 challenge [41,42,43].

Despite progress, real-world deployment of PPG remains constrained by the limited availability of ecologically valid datasets. While several public datasets provide multimodal recordings (e.g., PPG, ECG, accelerometry) [44,45,46,47,48,49,50], most were collected under controlled or sedentary conditions. For example, the MIT-BIH Polysomnographic Database [44] contains multiple physiologic signals recorded during sleep studies, while the Complex System Lab’s dataset [46] offers clinically annotated recordings for conditions such as sepsis, traumatic brain injury, and cardiac events. Only a few datasets capture recordings during physical activity or motion-rich contexts [47]. Jarchi and Casson [48] extended this effort by collecting synchronized PPG, ECG, and motion sensor data (accelerometers and gyroscopes) during activities such as walking, biking, and running. However, their recordings remain limited to structured indoor environments. In contrast, naturalistic settings, such as in-vehicle conditions, introduce a range of complex disturbances, including vibration, abrupt directional changes, fine motor activity, ambient light variation, and distributional signal drift. These real-world complexities are poorly represented in existing datasets, limiting model generalization and constraining reliable field deployment.

**Table 2:** Recommended current practices for PPG-derived signal processing for applications involving HR and HRV estimation.

Type	Norms	References
Sampling Rate	≥25 Hz is suggested.	[51]
Low Frequency Filtering	0.5 Hz to remove the direct current (DC) component below 0.1 Hz and respiratory component in the 0.1–0.5 Hz band.	[22]
High Frequency Filtering	10 Hz, corresponding to the position of fourth harmonics at 150 bpm, or third harmonics at 200 bpm.	[22]
Artifact Removal	- Decomposition-based: ICA, EMD, wavelet decomposition. - Adaptive filtering: RLS, LMS. - Deep neural network.	[26,27,28,29,30,31,32,33,34,35,52]
Fiducial Point Identification	- Zero crossing (change of slope sign). - Local maxima/minima with adaptive thresholding. - Deep neural network.	[39,40]
Signal Quality Index	- Machine learning with statistical features. - Deep neural network.	[36,37,38]
Feature Extraction	- PR: spectral peak tracking. - PRV: Extract the pulse-pulse interval and calculate features similar to HRV.	[41,42,43,53]

#### 1.1.3. Studies Using Pulse Rate Features as Natural Proxies for Heart Rate Features

Several studies have examined the validity of pulse rate (PR) and pulse rate variability (PRV) as proxies for ECG-derived HR and HRV. Under resting conditions, PPG-derived PR and PRV often show high agreement with ECG-based metrics, requiring minimal artifact correction [54,55]. For instance, Menghini et al. [56] evaluated PRV across resting, public speaking, and recovery tasks, finding strong agreement with ECG-HR at rest but diminished PRV accuracy with even minor movement. Similarly, Milstein and Gordon [57] reported divergence between PRV and ECG-HRV during dyadic conversations, highlighting sensitivity to behavioral and social engagement. In a six-week neurofeedback study, Schuurmans et al. [58] observed consistent correlations between PR and ECG-HR across sessions, whereas PRV exhibited variability during cognitively or emotionally demanding tasks. Van Voorhees et al. [59] compared wearable PPG and Holter ECG recordings during ambulatory activity and found low PRV reliability over short windows (1–5 min), reinforcing concerns about PRV robustness in field-based, non-resting contexts. In addition, peripheral PRV may capture physiological variance absent in ECG-derived HRV. Mejía-Mejía [60] reported that PRV responses to cold exposure differed from ECG-HRV, particularly at peripheral measurement sites, likely due to vascular or thermoregulatory effects. Because PPG captures the pulse wave after a temporal delay relative to cardiac depolarization, PRV may also reflect influences from the peripheral nervous system and local vascular dynamics [61]. Consequently, the use of PRV as a surrogate for HRV remains an area of active investigation, particularly in dynamic, in-vehicle or field-based environments where motion artifacts, signal degradation, and physiological noise complicate reliable estimation.

In summary, ECG directly measures cardiac electrical activity with clearly defined fiducial points and minimal motion sensitivity, whereas PPG reflects peripheral hemodynamic fluctuations that are inherently more susceptible to motion and sensor-contact variability. Recent developments in cardiovascular monitoring increasingly integrate PPG with auxiliary sensors (e.g., accelerometers) or employ deep-learning-based denoising and signal-quality modeling to mitigate these limitations. Together, these efforts mark a broader shift toward hybrid and context-aware processing frameworks that aim to balance interpretability, robustness, and ecological validity.

### 1.2. Present Study Objectives

Given the diversity of SQA approaches and BVP processing methods, there remains a critical need for a processing pipeline tailored specifically to the challenges of in-field PPG acquisition, particularly in dynamic, motion-rich environments such as vehicles. In these contexts, PPG signals are often degraded by motion artifacts, variable lighting, and sensor displacement, requiring more advanced processing than those used in traditional resting-state applications. Unlike most psychophysiological studies focused on sleep or sedentary conditions, emerging use cases involve light to moderate activity, such as passive vehicle riding or task engagement. To address these challenges, we propose a lightweight and interpretable PPG processing pipeline designed specifically for pulse rate monitoring under real-world dynamic conditions. Our approach prioritizes transparency and physiological plausibility, leveraging domain-informed signal features rather than relying on data-intensive deep learning models. This design achieves robust performance with reduced computational overhead and increased interpretability. The proposed pipeline is validated using a custom dataset collected from participants riding as passengers in an autonomous vehicle, a representative in-field setting that captures realistic sources of signal degradation. Distinct from prior multi-sensor fusion or black-box data-driven deep learning approaches, this study introduces an interpretable, single-channel SQA and spectral reconstruction pipeline calibrated against ECG-derived ground truth, specifically designed for motion-rich conditions in vehicle environments.

The primary aim of this study is to (1) assess the accuracy of PPG-derived HR and HRV estimates under dynamic, in-vehicle conditions, and determine which parameters can reliably serve as surrogates for ECG-derived features; (2) evaluate the effectiveness of various signal processing techniques, including signal quality thresholding, spectral reconstruction, and fiducial point detection, in recovering and improving PPG-derived metrics with respect to ECG references; and (3) explore trade-offs between maximizing useable data and preserving signal fidelity, and offer practical recommendations for the design and deployment of PPG signal processing pipelines in ecologically valid, real-world applications.

## 2. Methods

### 2.1. Data Source

A total of 114 trials (57 women and 57 men) were included for the current analysis. Data were collected as a part of a closed test-track study, with participants seated in the front passenger seat of an instrumented automated vehicle (AV). During the session, participants experienced 30 consecutive AV-generated acceleration-braking events while engaging in a visual task on a handheld device. Each event consisted of an acceleration from rest, a constant-speed cruising phase of variable duration, and a deceleration to a complete stop. These data were collected as part of a larger study on passenger motion sickness. The structured event sequence emulated stop-and-go traffic conditions and passenger engagement with handheld devices, enabling assessment of PPG signal resilience to motion artifacts during visually demanding, dynamic conditions.

Each participant wore two physiological monitoring devices to enable simultaneous ECG and PPG data collection. ECG signals were acquired using the Zephyr^TM^ Bioharness^TM^ 3 (Medtronic, Minneapolis, MN, USA) device, a wearable chest strap positioned immediately below the pectoral muscle, with the Biomodule aligned under the left arm. The BioHarness device employs a single-lead ECG configuration using conductive chest-strap electrodes and records R-R intervals at 250 Hz. Strap tension was standardized across participants using the integrated tension indicator loop to ensure consistent electrode contact. PPG signals, recorded as BVP, were collected using the Empatica E4 wristband (Empatica, Inc., Cambridge, MA, USA) worn on the non-dominant wrist just proximal to the wrist joint. The PPG sensor, embedded in the underside of the wristband, captured vascular signals at 64 Hz by measuring variations in reflected light, as blood volume changed with each heartbeat. In addition to PPG, the Empatica E4 recorded three-axis accelerometer data at 32 Hz, providing contextual information about movement during the in-vehicle protocol (see: https://www.empatica.com/en-int/store/e4-wristband/, accessed on 4 September 2025). It should be noted that, as of February 2025, the Empatica E4 and its associated software suite were formally discontinued (see: https://www.empatica.com/research/e4-sunset/, accessed on 4 September 2025). This discontinuation does not affect the integrity of data collected prior to the sunset period.

Prior to the in-vehicle session, participants completed two baseline recordings. First, a five-minute resting baseline was recorded while participants sat comfortably in a quiet indoor environment, following procedures outlined by Winslow et al. [62]. A second three-minute resting baseline was conducted inside the stationary vehicle. For both recordings, participants were instructed to remain still, avoid speaking, and minimize movement to ensure signal integrity. Immediately after the in-vehicle baseline, the experimental condition began. Both the Zephyr BioHarness and Empatica E4 devices recorded continuously throughout the ~20 min in-vehicle riding protocol.

Four trials were excluded from the final dataset due to quality issues: one due to absence of PPG data during the BioHarness recording period, one due to an early ECG termination, and two due to abnormal ECG waveforms lacking discernible beat structure. As a result, a total of 110 trials were included in the final analysis.

### 2.2. Data Processing

**Figure 2:**
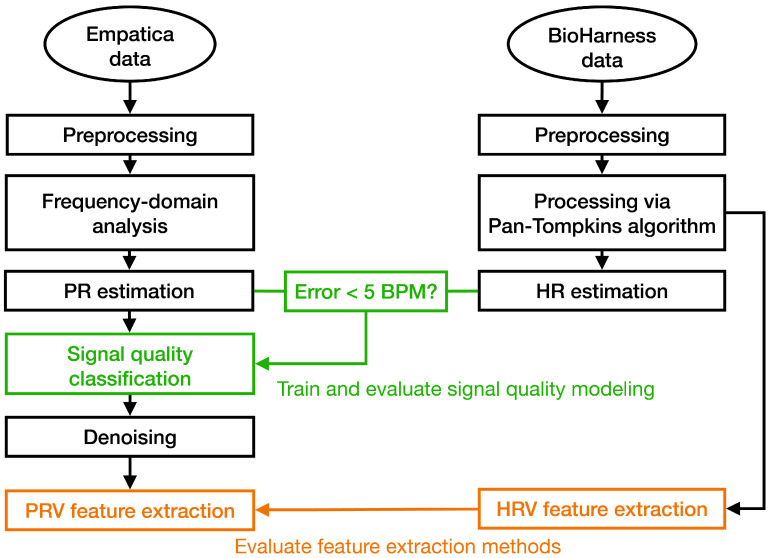
Data processing and evaluation flowchart. Empatica BVP-derived pulse rate (PR) estimates are compared with BioHarness ECG-derived heart rate (HR) estimates, with epochs showing <5 BPM error used to train the signal-quality classifier (green). PRV and HRV features (orange) are then extracted for method evaluation.

An overview of the data processing pipeline and the evaluation framework for assessing PR and PRV as surrogates for ECG-derived HR and HRV is shown in Figure 2. Detailed descriptions of the SQA modeling, signal reconstruction, fiducial point recognition, and feature extraction are provided in the following sections.

The processing of BioHarness ECG signals followed established gold-standard recommendations from the literature (see Table 1). Raw ECG signals were processed using the Pan–Tompkins algorithm, a widely used QRS-complex detection method based on derivative filtering, adaptive thresholding and integration [19]. The processed ECG data were then segmented into analysis epochs of varying lengths, ranging from 5 to 30 s. Within each epoch, interbeat intervals (IBI) were computed as the time difference between consecutive QRS detections. To improve data quality a two-step IBI outlier removal procedure was applied: (1) physiological range filtering: IBIs outside 300–1500 msec (equivalent to 40–200 bpm, the typical heart rate range in healthy adults) were excluded, consistent with established thresholds [15]; and (2) relative deviation filtering: IBIs deviating more than 30% from the mean of the four most recently accepted IBIs were also removed. The initial four IBIs were manually reviewed and selected to establish a reliable starting reference. Because ECG provides unambiguous fiducial points that remain stable under mild motion, it served as the gold-standard reference for all subsequent comparisons. PPG-derived PR and PRV metrics were then evaluated relative to these ECG-derived HR and HRV measures to quantify reliability.

Signal processing of the PPG data followed common practices outlined in the literature (see Table 3). The PPG signal, collected from the Empatica E4 wristband, was filtered using a fourth-order Butterworth bandpass filter with cutoff frequencies at 0.5 Hz and 10 Hz, to suppress baseline drift and high-frequency noise while preserving frequency components relative to heart rate, including up to the third harmonic of 200 bpm. Concurrent accelerometer data were filtered using a bandpass filter with cutoff frequencies of 0.025 Hz and 10 Hz to remove motion artifacts and DC bias. These preprocessed accelerometer signals were then incorporated into the signal-quality assessment model through a set of time- and frequency-domain motion features (e.g., amplitude variability, spectral power, and cross-spectral coupling with BVP) to quantify motion-induced contamination. Both PPG and accelerometer signals were segmented into analysis windows of 5 to 30 s, synchronized with the ECG-based epochs. Within each epoch, a fast Fourier transform (FFT) was performed to extract frequency-domain features with a 1 bpm resolution. An SQA classifier was applied to each epoch, trained using binary labels (e.g., usable vs. unusable) determined by the discrepancy between PR, estimated from the dominant spectral peak in the PPG signal, and the reference HR derived from ECG. Epochs labeled as unusable were excluded from subsequent analyses. The remaining PPG epochs underwent frequency-domain signal reconstruction using a notch-filter-based denoising procedure to further enhance waveform quality. From these cleaned signals, PRV features were extracted using the same computational framework applied to ECG-derived HRV, enabling direct comparison. Finally, ablation studies were conducted to examine the relative contribution of each component, SQA and signal reconstruction, by systematically removing these steps and evaluating the trade-offs between data retention and feature accuracy when using PPG-derived metrics as surrogates for ECG-based HR and HRV.

#### 2.2.1. Signal Quality Assessment

**Figure 3:**
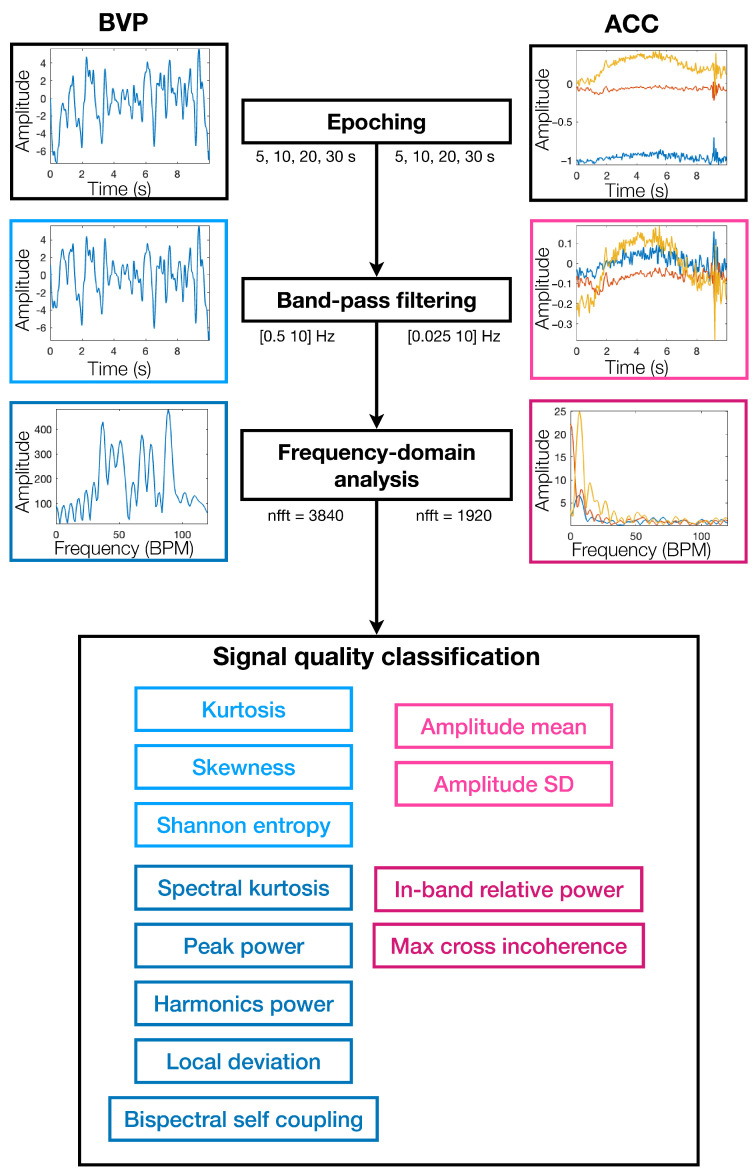
Procedures of the signal quality assessment (SQA) classification model. Left: Blood volume pulse (BVP) signal processing. Right: Accelerometer signal processing. Bottom: Extracted features from both modalities at different stages used as input to the SQA classifier. Blue elements denote features derived from the BVP signal, and pink elements denote features derived from the accelerometer signal. Lighter hues represent the original time-domain signals, and darker hues represent derived or frequency-domain representations.

We developed an SQA classification model using a comprehensive set of time- and frequency-domain features derived from both PPG-derived BVP and accelerometer signals. This model builds upon and extends methods proposed in prior literature [30,36,37,38]. Figure 3 illustrates the full signal-quality assessment (SQA) pipeline, including preprocessing, frequency-domain analysis, and feature extraction for both BVP and accelerometer signals. A detailed list of features incorporated in the SQA model is provided in Table 3. Among the morphological features, we included kurtosis, skewness, and Shannon entropy of the BVP signal, as these metrics are widely recognized as reliable indicators of signal quality [63,64]. In the frequency domain, we extracted the relative power of the estimated heart rate frequency (HRF), specifically the dominant spectral peak within the HR band, and its second and third harmonics, to characterize the spectral sharpness and prominence of the HR-related peaks [65]. To identify abrupt disruptions caused by motion or noise, we computed the absolute difference between the current HRF and the 1 min median HRF. Following prior work [63], we also included frequency-domain kurtosis of the BVP signal and bispectral self-coupling, which reflect spectral sparsity and cross-frequency coupling, respectively. To capture motion-related interference, we derived the mean and standard deviation of the accelerometer signal magnitude, along with the relative power of the accelerometer signal within the heart rate band and the maximum cross-bicoherence between the accelerator and PPG signals, quantifying the extent of motion contamination in the frequency domain [66]. Because it relies on a compact set of twelve explicitly defined signal-quality and motion-related features, the proposed model remained computationally lightweight, requiring only standard feature extraction and a single classification step, while maintaining direct physiological interpretability for each feature.

Epoch labeling for training the SQA classifier was performed automatically. Epochs were labeled as “acceptable” if the BVP signal contained the dominant spectral peak that matched the ECG-derived HR within ±5 bpm tolerance. This frequency-domain approach enabled a preliminary signal-quality check, reducing reliance on morphological pulse detection and allowing a more flexible thresholding for identifying usable epochs. These quality labels also served as a basis for downstream signal reconstruction, discussed in the subsequent section.

**Table 3:** Feature extraction of PPG-derived and accelerometer sensor measures for the SQA classification model.

Source	Feature	Description	References
BVP time domain	Kurtosis	Scaled version of the fourth moment of the PPG distribution, representing the tailedness of the PPG signal distribution. Measures peakedness of the waveform; high kurtosis may indicate clean, sharp pulse waves	[30]
	Skewness	Measure of the asymmetry of the PPG signal around zero. Describes waveform asymmetry; deviations from symmetry may signal noise or distortion	[30]
	Shannon entropy	Measure of the disorder in the PPG signal probability distribution. Quantifies signal complexity; higher entropy may suggest irregularity due to noise	[30]
BVP frequency domain	Spectral kurtosis	Scaled version of the fourth moment of the PPG spectral distribution, representing the tailedness of the PPG frequency-domain signal. Detects spectral sparsity; flatter spectra may indicate noise or artifact	[30]
	Relative power of dominant peak	Power ratio of the dominant peak in the PPG spectrum compared to the total power. Power of the peak frequency in the heart rate band; used to confirm signal periodicity	[67]
	Relative power of harmonics	Power ratio of the 2nd and 3rd harmonics of the PPG spectral dominant peak compared to the total power. Power in harmonic components; supports waveform integrity checks	[67]
	HRF deviation from moving median	Absolute difference between the spectral peak of the current PPG epoch and the median spectral peak of the nearest 1 min segment. Measures abrupt change in pulse frequency; used to detect transient noise	
	Bispectral self-coupling	Number of self-coupling events among the three most prominent peaks (f0, f1, f2) in the diagonal slice of the bispectrum. Assesses cross-frequency coupling around HRF; reduced coupling may signal distortion	[68]
Accelerometer time domain	Amplitude mean	Average magnitude of the accelerometer data. Indicates overall movement intensity; elevated values may suggest a potential motion artifact	[38]
	Amplitude SD	Standard deviation of the accelerometer magnitude. Captures motion variability; high standard deviation often correlates with motion-induced noise	[38]
Accelerometer frequency domain	Maximal cross-bicoherence to PPG	Maximum bicoherence between the PPG signal and the accelerometer data from the x-, y-, or z-axis. Measures motion energy overlapping the HR band; used to detect confounding artifact sources	[66]
	Relative power of heart rate frequency band	Relative power of the [2/3, 10/3] Hz band, corresponding to the heart-rate frequency band ranging from 40 BPM to 200 BPM. Estimates nonlinear coupling between motion and the pulse signal; high values imply motion contamination	Inspired by [69]

A total of 12 features were computed for each epoch. To optimize feature selection we applied the minimum redundancy maximum relevance (mRMR) algorithm [70], which uses mutual information to identify features that maximize predictive relevance while minimizing inter-feature redundancy. A series of models was iteratively trained by progressively including top-ranked features, from the single highest-ranked feature up to the full feature set. To assess classification performance, we trained models using various classifiers, including Support Vector Machine (SVM), Linear Discriminant Analysis (LDA), Naive Bayes (NB), and Logistic Regression. In order to evaluate the influence of epoch duration, separate models were trained for 5, 10, 20, and 30 s epochs. For each epoch length, participant data were segmented into minute-long intervals, with 80% used for training and 20% randomly sampled for testing.

Due to the labeling method, most epochs were classified as “acceptable”, resulting in a class imbalance. To mitigate this, we performed a grid search to optimize the misclassification cost ratio between the majority and minority classes (range: 1–10). For each model type and epoch length, the optimal number of features and class weight ratio were determined by maximizing the minimum of sensitivity and specificity using five-fold cross-validation.

#### 2.2.2. Signal Reconstruction

**Figure 4:**
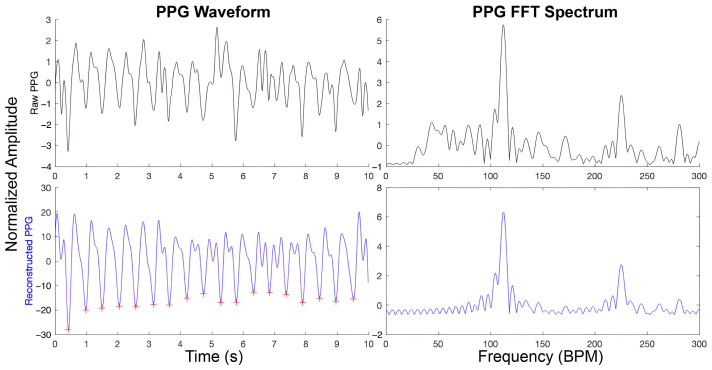
An illustration of PPG signal reconstruction using spectral peak information. The first row shows the original PPG waveform in the time-domain (left) and frequency-domain (right). The second row presents the reconstructed signal, with only information corresponding to the heart rate frequency and its harmonics retained. Red plus signs indicate the detected systolic peaks (beat locations) in the reconstructed waveform.

Signal reconstruction was applied to all epochs classified as acceptable by the SQA modeling using a targeted notch-filtering approach adapted from [71]. Unlike traditional Independent Component Analysis (ICA)-based denoising, which requires multiple PPG sources, this method is optimized for a single-channel wrist-worn PPG signal, such as those recorded by the Empatica E4. While adaptive filtering and wavelet decomposition are widely used for noise suppression, they typically depend on the availability of a reference signal correlated with motion artifacts. When this assumption is violated, such methods risk attenuating physiologically meaningful components of the PPG waveform. Motion-based denoising strategies can be effective during high-intensity exercise, where motion dominates the noise spectrum, but are less suitable for passive dynamic environments like vehicle rides, where artifact sources are more heterogeneous and often uncorrelated with gross movement. Accordingly, we adopted a targeted frequency-domain reconstruction method that leverages the reliability of estimated heart rate frequency (HRF) and its second harmonic. The raw PPG signal was passed through a cascade of notch filters centered at the dominant HRF and its second harmonic. Each notch filter was implemented as a 4th-order Butterworth band-stop IIR filter with half-power frequencies set at ±20% around the estimated HRF and its harmonic. At a 64 Hz sampling rate of the E4 device, this configuration yielded rejection bands of approximately 0.2–0.4 Hz for typical adult HR ranges (1–2 Hz), effectively attenuating motion-related peaks near the HRF while preserving the surrounding physiological content. The filtered signal, representing noise localized to the HRF and harmonic bands, was subtracted from the raw PPG waveform to reconstruct the cleaned signal. This approach preserves physiologically relevant components while suppressing confounding, in-band noise not associated with the dominant cardiac rhythm. Figure 4 illustrates representative PPG waveforms before and after this reconstruction procedure.

#### 2.2.3. Fiducial Point Detection and Evaluation

Following signal thresholding and reconstruction, we applied two fiducial point detection methods to identify pulse peaks and onsets in the PPG time series. The first method, Multi-Scale Peak and Trough Detection (MSPTD), is widely used in pulse detection research. MSPTD segments the signal, generates a local maxima scalogram across multiple temporal scales, identifies the most relevant scale for pulse detection, and refines the peak and onset locations within a narrow time window [72]. This method has been shown to outperform several competing algorithms across diverse datasets in previous studies (see Section 1).

In addition to MSPTD, we employed a method developed at the University of Iowa’s Driving Safety Research Institute, named Multivariate Normal Density Estimation and Imputation (MNDEI). Beginning with the E4 IBI data, a multivariable normal probability density function was built using the BVP and its second derivative at determined IBI points, as well as the standard deviation of BVP in a one-second window around IBI points. To better capture normal distributions, the log of all three variables was used. This density function was used to detect other BVP points that fell within its distribution. Further refinement was achieved by (a) removing abnormally small IBIs and abnormally isolated pulses, (b) imputing missing IBI samples using available local BVP minima, and (c) repeating the removal of abnormally small IBIs and isolated pulses.

To evaluate how different combinations of processing methods affect the accuracy of BVP-derived HR and HRV features, we conducted a comparative analysis across six configurations:

(1) Raw signal + MSPTD.
(2) Raw signal + MNDEI.
(3) SQ-threshold signal + MSPTD.
(4) SQ-threshold signal + MNDEI.
(5) SQ-threshold signal + reconstruction + MSPTD.
(6) SQ-threshold signal + reconstruction + MNDEI.

For each condition, we computed heart rate and a standard set of time-domain and frequency-domain HRV features from both ECG and PPG signals. ECG-derived HRV was treated as the ground truth reference. HRV metrics were calculated using the PhysioNet Cardiovascular Signal Toolbox [73], with a 5 min window and 30 s step size applied across the trial data. As per prior recommendations, ECG-derived epochs with more than 15% missing data were excluded from analysis, particularly for high-frequency features, which are sensitive to data gaps [74]. In total, seven trials were excluded due to insufficient ECG coverage, resulting in 2612 valid ECG epochs. For PPG-derived epochs, a 50% missing-data threshold was adopted. Stricter thresholds (e.g., 15% or 25%) resulted in no process pipeline achieving more than 40% epoch coverage, significantly limiting analysis viability. To evaluate the agreement between ECG- and PPG-derived metrics, we conducted the following: Pearson correlation to assess linear relationships, Cliff’s δ to assess distributional effect sizes and agreement, and Bland–Altman analysis to evaluate the bias and limits of agreement (LOA). These analyses provide insight into the relative performance of different fiducial point detection and signal processing pipelines under dynamic, in-vehicle conditions.

## 3. Results

### 3.1. Signal Quality Assessment Classification Model: Performance

A detailed summary of the cross-validation results for the SQA classification models is presented in Table 4. Among the evaluated models, the classifier trained on 10 s epochs outperformed those trained on shorter (5 s) or longer (20 s and 30 s) time windows. This model achieved the highest balance between accuracy, specificity, and sensitivity. The best performance was observed using an SVM classifier with eight features selected via mRMR and a misclassification cost ratio of 3, resulting in a minimum specificity and sensitivity of 0.884 during five-fold cross-validation. This configuration was selected as the final model and subsequently evaluated on the 20% participant-level holdout test set. On this unseen data, the model achieved an overall accuracy of 0.888, a specificity of 0.892, a sensitivity of 0.887, and an AUC of 0.960. These results demonstrate the model’s robustness and generalizability in distinguishing usable versus unusable PPG epochs for subsequent analysis.

### 3.2. Comparison of Signal Processing Methods for PRV Features

#### 3.2.1. Percentage of Valid Epochs

**Figure 5:**
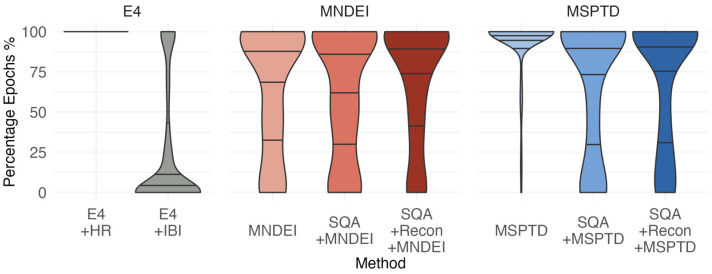
Violin plot illustrating the distribution of the percentage of epochs retained for HRV analysis across trials, grouped by processing method. For all methods except the E4 HR, only epochs where at least 50% of the IBIs are available are used for calculating heart rate and heart rate variability metrics. For the E4 HR method, only the mean heart rate is analyzed without IBI data, so all epochs are included regardless of IBI coverage.

Figure 5 presents a violin plot illustrating the distribution of the percentage of valid epochs retained for HRV analysis trials and processing methods. This analysis quantifies the proportion of epochs that yielded usable HRV data, offering insight into the data retention trade-offs for each method. The Empatica E4’s native HR output, provided at 1 Hz resolution, is smoothed and continuous, regardless of the confidence indicator in the output. For this signal, the average heart rate was computed as the cumulative mean of the E4 HR output within each epoch. No HRV features were derived from the native E4 HR, due to its smoothing and preprocessed nature.

For all other methods, including those employing fiducial point detection algorithms (e.g., MSPTD and MNDEI), both HR and HRV metrics were computed, and only epochs with ≤50% missing data were retained for analysis. The E4 IBI output, used without manual cleaning, showed poor data retention: in 50% of trials, fewer than 12.5% of epochs were considered valid.

The use of the MNDEI method significantly improved epoch inclusion, while MSPTD yielded the highest number of valid epochs across trials. As expected, the application of SQ thresholding reduced the number of retained epochs, as its purpose is to exclude segments that do not provide reliable pulse information. However, the inclusion of signal reconstruction following SQ thresholding led to modest recovery in valid epoch count, as the cleaned signals enabled pulse detection in previously borderline or noisy segments. Overall, the combined application of SQ thresholding and signal reconstruction resulted in stable performance, with a median of approximately 75% valid epochs retained across trials.

#### 3.2.2. Pearson Correlation

**Figure 6:**
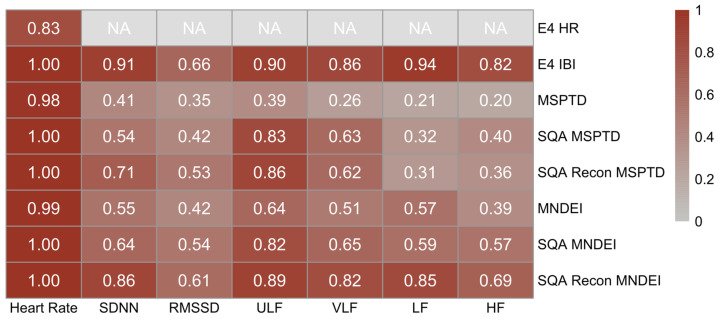
Pearson correlation between ECG-derived HRV and PPG-derived PRV metrics. The heatmap matrix of Pearson correlation coefficients across multiple methods. Cells labeled “NA” indicate HRV metrics that are not available as outputs from Empatica E4. All Pearson correlation tests have a *p*-value of < 0.001.

Using the accepted epochs, as defined in the prior selection, Figure 6 presents the Pearson correlation coefficients between ECG-derived HR and HRV metrics and their corresponding PPG-derived PR and PRV metrics, computed across the different processing pipelines. A consistently strong correlation in HR is observed for all processing methods, exceeding the correlation observed for the native E4 HR output. Despite the low percentage of valid epochs noted previously, the E4 IBI output shows strong correlations (r > 0.8) with most HRV features, except for RMSSD, which appears to be more sensitive to signal noise and preprocessing variability. The MSPTD method, although associated with a high valid epoch percentage, shows weak correlations (r ≤ 0.3) for frequency-domain HRV metrics (VLF, LF, HF) and moderate correlation (r ≤ 0.5) for time-domain metrics (SDNN, RMSSD) and ULF. The application of SQ thresholding prior to MSPTD slightly improves correlations, while the combined application of SQ thresholding and reconstruction further enhances performance. Similar trends are observed with MNDEI. When used without preprocessing, MNDEI achieves modest correlations; however, when paired with SQ thresholding and reconstruction, it demonstrates the strongest agreement with ECG-derived metrics among the processing methods. Specifically, this full method (SQ thresholding + reconstruction + MNDEI) yields the strongest correlations for all HRV variables except RMSSD and HF, where results remain moderate but improved compared to other methods.

#### 3.2.3. Cliff’s δ Effect Size

**Figure 7:**
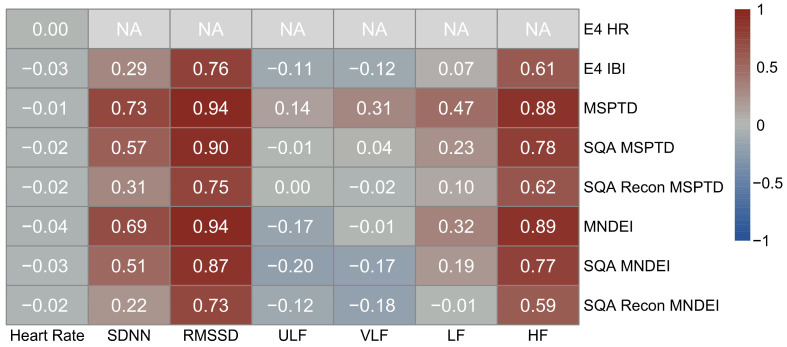
Cliff’s δ effect size between ECG and PPG-derived HRV metrics. Heatmap matrix displaying Cliff’s δ values for each HRV metric (HR, SDNN, RMSSD, ULF, VLF, LF, HF) across multiple PPG processing methods. Cells labeled “NA” indicate HRV metrics that are not available as outputs from Empatica E4. Values indicate the degree of distributional non-overlap, where δ ≥ 0.474 denotes a large effect size.

Figure 7 presents Cliff’s δ effect sizes, quantifying the degree of distributional non-overlap between PPG-derived metrics and ECG-derived ground truth across different signal processing methods. This nonparametric measure complements Pearson correlation by evaluating the magnitude and direction of bias in estimated HRV metrics. Among all the variables examined, RMSSD and HF power are particularly prone to systematic overestimation by PPG-derived measures, with all methods, including E4 IBI output, yielding large effect sizes (δ ≥ 0.474). SDNN and LF also exhibit overestimation, though to a more moderate degree. In contrast, ULF and VLF show a tendency toward underestimation, depending on the processing method. For all HRV variables except RMSSD and HF, the magnitude of distributional divergence is reduced when applying SQ thresholding and reconstruction, suggesting these steps improve agreement with ECG. However, for RMSSD and HF, even the most refined processing methods fail to reduce the effect size below the large non-overlap threshold, highlighting the persistent difficulty in recovering these parasympathetic-sensitive metrics from wrist-worn PPG.

#### 3.2.4. Bland–Altman Analysis

**Figure 8:**
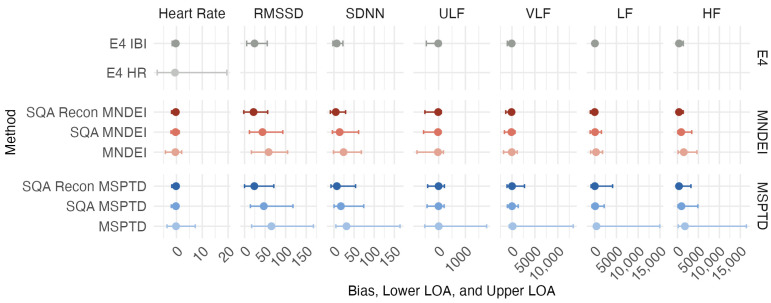
Bland–Altman analysis of PPG- and ECG-derived HRV metrics. Median bias and nonparametric 95% limits of agreement (LOA) between HRV and PRV across the processing methods. The figure shows the median difference (PRV-HRV) as a central dot, with error bars representing the 95% LOAs.

A Bland–Altman analysis was conducted to assess the agreement between PPG- and ECG-derived HRV features. Prior to analysis, the Kolmogorov–Smirnov test was applied to all feature distributions, confirming that none of the HRV variables followed a normal distribution. As a result, bias was calculated using the median difference between PPG-derived PRV and ECG-derived HRV values. The limits of agreement (LOAs) were defined as the 2.5th and 97.5th percentiles of the paired differences. The resulting bias and LOAs for each processing method are presented in Figure 8. Among the manual processing methods, both MSPTD and MNDEI demonstrated less median bias when SQ thresholding and reconstruction were applied. MSPTD combined with SQ control also led to a narrower spread in bias, indicating improved precision. Notably, when MNDEI was combined with both SQ thresholding and signal reconstruction, the resulting bias values were comparable to those observed for the E4 IBI output, while still retaining substantially more valid epochs per trial. This result suggests that preprocessing steps not only improve agreement with ground truth but also preserve a higher volume of analyzable data, which is critical for in-field applications.

#### 3.2.5. Summary of Signal Processing Methods for PRV Features

To facilitate direct comparison across processing approaches, we summarize the average performance of each pipeline in Table 5. The table reports the percentage of valid epochs retained, Pearson correlation coefficients, Cliff’s δ effect sizes, and median bias values for both HR and HRV metrics relative to ECG-derived ground truth. The results show that while manufacturer-processed E4 IBI data yield the highest HR correlation, they capture fewer valid epochs (22.7%). Manual pipelines such as MSPTD and MNDEI substantially improve data retention but introduce greater HRV bias. Incorporating the signal-quality assessment (SQA) threshold and spectral reconstruction increases HRV correlation and reduces bias, most notably for the SQA Recon MNDEI method, which achieved a Pearson r = 0.79 and reduced bias to 41.66% while maintaining approximately 70% valid data. These outcomes highlight the advantage of coupling lightweight SQA filtering and reconstruction steps to enhance PPG reliability under dynamic, in-vehicle conditions.

## 4. Discussion

This study evaluated the accuracy and limitations of PPG for estimating HR and HRV under dynamic conditions, using ECG-derived metrics as the gold standard. While PPG-based sensors are increasingly favored in wearable technology due to their comfort, affordability, and ease of integration, our findings reveal that their reliability as a surrogate for ECG varies substantially across cardiovascular features and environmental context.

Our results demonstrate that PPG can reliably estimate PR in dynamic in-vehicle conditions, irrespective of the signal processing pipeline employed. In contrast, PRV, a substitute for ECG-derived HRV, exhibits considerably lower consistency and accuracy. Although some time-domain and frequency-domain PRV metrics showed moderate correlation with their ECG-derived counterparts, high-frequency metrics, such as RMSSD and HF power, were notably unreliable and prone to overestimation.

The discrepancies between PRV and HRV stem from both technical and physiological factors. Technically, motion artifacts and ambient noise distort the PPG waveform, particularly in mobile environments, impeding accurate detection of fiducial points. Physiologically, pulse transit time delays between the heart and peripheral measurement sites (e.g., wrist) introduce additional variability absent in ECG-derived signals. Moreover, vascular dynamics and local autonomic responses at the peripheral site can further distort high-frequency HRV components. These factors collectively contribute to significant deviations in PRV, particularly in metrics sensitive to short-term autonomic fluctuations.

A key challenge identified in this study is the trade-off between data quantity and signal quality. Increasing the number of epochs maximizes data availability but also raises the risk of including noisy or artifact-laden segments. To address this, we applied SQ thresholding and frequency-domain signal reconstruction. These methods improved PRV extraction by filtering out unreliable epochs and recovering usable signal components. Applying the SQ classifier prior to reconstruction reduced the proportion of motion-contaminated epochs from roughly 95% valid (MSPTD) to 67%, yet substantially improved HRV accuracy: the mean Pearson r increased from 0.31 to 0.53 and Cliff’s δ decreased from 0.58 to 0.42. When frequency-domain reconstruction was subsequently applied, the valid-epoch percentage recovered to 70% and the HRV correlation further improved to r = 0.57 with δ = 0.29, halving the average bias (from 110.83% to 55.90%). A similar pattern held for the MNDEI pipeline, where the full SQA + reconstruction configuration yielded the strongest HRV agreement (r = 0.79, δ = 0.21) while maintaining ~70% of analyzable epochs. These results demonstrate that lightweight quality control and spectral reconstruction substantially mitigate motion artifacts and enhance reliability without increasing computational cost. However, even the most effective processing methods could not fully eliminate motion-induced noise. Moreover, aggressive thresholding, while improving precision, significantly reduced the number of usable epochs, particularly for HRV analysis. This highlights the delicate balance between accuracy and data coverage in PPG signal processing.

Our findings advance the literature by demonstrating that a lightweight, single-channel pipeline that combines feature-based signal-quality filtering, narrow-band spectral reconstruction, and robust peak detection (MNDEI) can substantially narrow the longstanding performance gap between wrist-worn PPG and gold-standard ECG in mobile, real-world contexts. Previous studies have consistently shown that while pulse rate estimates from PPG align well with ECG under resting conditions, PRV performance deteriorates markedly even with mild movement or speech. In such dynamic conditions, PRV correlations with ECG-derived HRV often fall below 0.4, with data-loss rates exceeding 60% during ambulatory tasks or treadmill exercise [41,56,57,58,59]. Recent advances in motion-compensation, such as multi-sensor adaptive filtering [26,27,28,29,31] and deep-learning-generative models [34,35], have improved PRV estimation but typically incur significant trade-offs. These methods require additional data channels, require large and labeled datasets for training, and often lack interpretability. Moreover, many methods fail to define objective thresholds for unrecoverable signals, allowing poor quality segments to persist after post-processing. In contrast, our combined SQ-threshold + reconstruction approach achieved a median of ~75% of usable epochs, while elevating PRV correlations for time-domain and low-frequency HRV metrics into the moderate-to-strong range (r = 0.6–0.8). Importantly, this was accomplished without sacrificing bias, which remained comparable to Empatica’s processed IBI stream. By replacing subjective manual annotation [36,37,38] with objective, ECG-derived ground-truth labeling, our approach eliminates inconsistency in SQA classification and enables reliable, reproducible filtering of low-quality data. These findings underscore that well-calibrated, interpretable preprocessing pipelines can offer meaningful performance gains without the computational overhead of black-box or multi-sensor solutions. As such, this work enhances the practical utility of wrist-based PPG for estimating cardiovascular metrics in in-vehicle and other ecologically valid, dynamic environments.

These findings have important implications for the application of PPG in wearable health monitoring, particularly in field contexts. It is important to note, however, that the present study was conducted under predominantly cyclical longitudinal accelerations, with only a single controlled lateral maneuver per trial. As such, the motion profiles differ from those encountered in more naturalistic driving, which typically includes frequent turns, variable terrain, and passenger interactions. This may limit the generalizability of the current results. Future studies should incorporate a broader range of real-world driving dynamics to better characterize motion-induced artifacts and assess the robustness of PPG processing pipelines under more diverse conditions. Continued work is needed to develop more robust noise-reduction strategies, including context-aware filtering, sensor fusion with accelerometry, deep learning-based denoising, and hardware innovations aimed at improving signal fidelity during motion and in-vehicle operation.

Finally, our study underscores the need for standardized protocols for PPG acquisition and processing for dynamic conditions, such as in-vehicle environments. Variability in device placement, sensor specifications, and preprocessing methods remains a significant barrier to cross-study comparability and broad clinical adoption. Establishing community-accepted benchmarks and open datasets will be essential for advancing the reproducibility and reliability of PPG-based physiological monitoring.

## 5. Limitation

This study used the Empatica E4 wristband as the source of all PPG recordings. Although widely adopted in prior research, the E4 and its software suite were discontinued in February 2025, and several evaluations have noted limitations in its PPG module, including reduced accuracy during movement, sensitivity to environmental noise, and relatively low sampling rates (64 Hz for PPG; 32 Hz for accelerometry). These constraints may have affected the fidelity of pulse-derived features in this study, particularly high-frequency HRV metrics that depend on precise waveform morphology. While the discontinuation does not affect the integrity of the present dataset or the performance gains achieved when applying the proposed pipeline to raw E4 PPG output, it may limit direct replication. Differences across newer wrist-worn PPG devices, such as optical design, sampling rate, and firmware, should be considered when applying or extending the present signal-quality assessment and reconstruction pipeline.

## 6. Conclusions

In conclusion, PPG remains a valuable tool for comfortable and low-cost heart rate monitoring, but its use as a reliable surrogate for HRV, particularly in dynamic environments, is still limited. Our results show that while signal processing improvements can enhance HR estimates and modestly improve PRV accuracy, substantial challenges remain for high-frequency HRV metrics. Future advancements in signal quality assessment, reconstruction, and denoising, coupled with standardization efforts, are critical to unlocking the full potential of PPG for real-world, wearable health applications.

## Figures and Tables

**Table 1:** Established ECG signal processing methods.

Type	Standards	References
Sampling Rates	Optimal: 250–500 Hz. Minimum: 100 Hz with parabolic interpolation.	[16]
Low Frequency Filtering	Optimal: 0.05 Hz.	[17]
High Frequency Filtering	Adults: 150 Hz. Children: 250 Hz.	[14,18]
Fiducial Point Identification	Use a well-tested algorithm (derivative + threshold, template, or correlation method) to locate a stable, noise-independent reference point.	[19]
Feature Extraction	- Heart rate. - Time-domain HRV: SDNN, RMSSD, pNN50. - Frequency-domain HRV: ULF, VLF, LF, HF, LF/HF. - Nonlinear HRV: SD1 and SD2, approximate entropy, sample entropy, MSE, DFA.	[20]

**Table 4:** Cross-validation performance of signal quality assessment (SQ) classification models. Bolded entries indicate the best-performing classifier, as determined by the highest accuracy and balanced sensitivity/specificity.

Epoch (s)	Classifier	Optimal Feature N	Optimal Cost	Accuracy	Specificity	Sensitivity	Min of Specificity and Sensitivity
5	Logistic	13	5	0.874	0.885	0.871	0.871
	SVM	10	5	0.868	0.888	0.862	0.862
	NB	9	9	0.872	0.874	0.872	0.872
	LDA	9	10	0.877	0.876	0.878	0.876
10	Logistic	4	3	0.890	0.878	0.892	0.878
	**SVM**	**8**	**3**	**0.885**	**0.884**	**0.885**	**0.884**
	NB	6	2	0.870	0.873	0.869	0.869
	LDA	4	7	0.890	0.881	0.892	0.881
20	Logistic	12	2	0.810	0.811	0.810	0.810
	SVM	7	2	0.799	0.824	0.789	0.789
	NB	4	2	0.805	0.804	0.805	0.804
	LDA	9	3	0.798	0.844	0.780	0.780
30	Logistic	1	1	0.750	0.699	0.780	0.699
	SVM	1	1	0.745	0.739	0.749	0.739
	NB	1	1	0.721	0.715	0.725	0.715
	LDA	1	1	0.747	0.715	0.765	0.715

**Table 5:** Average performance across signal-processing pipelines. Cells labeled “NA” indicate HRV metrics that are not available as outputs from Empatica E4 and therefore cannot be included in the comparison analysis.

Method	Valid Epoch	Heart Rate			Heart Rate Variability		
		Pearson r	Cliff’s δ	Bias	Pearson r	Cliff’s δ	Bias
E4 HR	100.00%	0.83	−0.01	−0.77%	NA	NA	NA
E4 IBI	22.70%	1.00	−0.03	−0.37%	0.85	0.25	47.43%
MSPTD	94.70%	0.98	−0.01	−0.25%	0.31	0.58	205.47%
SQA MSPTD	66.80%	1.00	−0.02	−0.30%	0.53	0.42	110.83%
SQA Recon MSPTD	70.30%	1.00	−0.02	−0.25%	0.57	0.29	55.90%
MNDEI	64.30%	0.99	−0.04	−0.60%	0.51	0.44	143.84%
SQA MNDEI	60.90%	1.00	−0.03	−0.49%	0.64	0.33	93.44%
SQA Recon MNDEI	69.50%	1.00	−0.02	−0.34%	0.79	0.21	41.66%

## Data Availability

The data that support the findings of this study are available from the corresponding author upon reasonable request.
